# Challenges affecting prompt access to adequate uncomplicated malaria case management in children in rural primary health facilities in Chikhwawa Malawi

**DOI:** 10.1186/s12913-019-4544-9

**Published:** 2019-10-22

**Authors:** Larissa Klootwijk, Anthony Emeritus Chirwa, Alinune Nathanael Kabaghe, Michele van Vugt

**Affiliations:** 1 Academic Medical Centre, University of Amsterdam, Redemptoristenpad 18, 5211XR, Den Bosch, Amsterdam, Netherlands; 2 Training and Research Unit of Excellence, College of Medicine, University of Malawi, Blantyre, Malawi; 3 Department of Internal Medicine and Infectious Diseases, Academic Medical Centre, University of Amsterdam, Amsterdam, Netherlands

**Keywords:** Malaria, Health systems, Malaria case management

## Abstract

**Background:**

Reducing the burden of malaria highly depends on access to prompt and effective malaria diagnosis and treatment. The aim of this study was to identify challenges affecting prompt access to effective uncomplicated malaria case management in children below 10 years old in rural primary health care facilities in Malawi.

**Methods:**

A cross sectional health facility survey was conducted in six primary health facilities in Chikhwawa district, Malawi. Officers-in-charge of health facilities were interviewed on availability of staff, supplies and drugs. All consecutive children presenting at the facility with fever or suspected malaria, aged 6 months to 10 years old, were eligible to participate in exit interviews. Exit interviews with participants’ guardians assessed duration of illness, demographic information and distance travelled. Adherence to recommended malaria case management guidelines included performing malaria rapid diagnostic tests (mRDTs) in children with fever or suspected malaria and prescribing recommended weight-based dose of artemether-lumefantrine (AL) when mRDT was positive. Multivariate logistic regression was used to determine factors associated with prompt care seeking within 24 h of onset of illness.

**Results:**

Health facilities were staffed by at least two health workers. Of 265 children screened, nine were excluded due to severe illness. Twenty-one percent of children presenting at a health facility with fever were not tested for malaria. Adherence to positive and negative mRDT results for those tested was 99.4, 95% CI [98.1–100] and 97, 95% CI [88.9–100], respectively. AL was prescribed as recommended by weight in 152 children (92.2%). Temporary stock outs of AL occurred in five of six facilities. In total, 146 (57, 95% CI [52.7–64.1]) guardians of patients sought care within 24 h after fever onset. Children aged 5 to 10 years were less likely to present within 24 h of fever onset than children below 5 years of age (unadjusted odds ratio 0.40, 95% CI [0.2–0.7]).

**Conclusion:**

Adherence to malaria diagnosis and treatment guidelines was high. However, delayed care seeking and stock outs may affect prompt and effective malaria case management. Further qualitative work is required to determine, and address factors associated with delay in care seeking for fever.

## Background

Despite several control measures, malaria remains a major public health problem in Malawi, with an estimated incidence of 3.3 million cases and 7200 deaths reported in 2015 [1]. One important strategy to reduce the burden of malaria is improved access to prompt and effective malaria diagnosis and treatment. Delay in accessing malaria treatment may lead to development of severe disease, continued disease transmission in the community and death [2]. Prompt diagnosis and treatment with artemisinin-based combination therapy (ACT) reduces gametocytaemia which reduces the risk of transmission and severity of disease [3, 4]. However, diagnosis and treatment of malaria and prompt health care seeking behaviour for fever continues to be a challenge especially in rural areas with poor health systems [5, 6].

Access to malaria case management comprises availability, acceptability, affordability and adequacy of health services [7]. Availability of functional health facilities, competent health workers, medical equipment and medical drugs and supplies are all required to provide appropriate malaria case management [2]. For malaria case management, diagnostic tests and recommended drugs need to be available. Additionally, health workers need to adhere to recommended treatment guidelines. Adherence to current WHO malaria guidelines and Malawi Standard treatment guidelines [2, 8] by health workers has been a challenge in rural Malawi [9–12]. A national health facility survey in 2014 in Malawi estimated that 1.5 million of 4.4 million malaria patients seen in public health facilities were not receiving appropriate treatment, with 2.7 million patients without clinical malaria inappropriately prescribed ACTs [10]. However, a health facility survey in southern Malawi in 2015 showed adherence to malaria rapid diagnostic test (mRDT) result was above 90% for both positive and negative mRDT results [12].

The purpose of this study was to identify challenges affecting prompt access to adequate uncomplicated malaria case management in children below 10 years old. We evaluated the adherence of health workers to malaria treatment guidelines and identified its associated factors. We also determined caregiver’s promptness to seek care and factors which were associated with delay in seeking care for fever within 24 h of onset.

## Methods

### Study design and setting

**Fig. 1:**
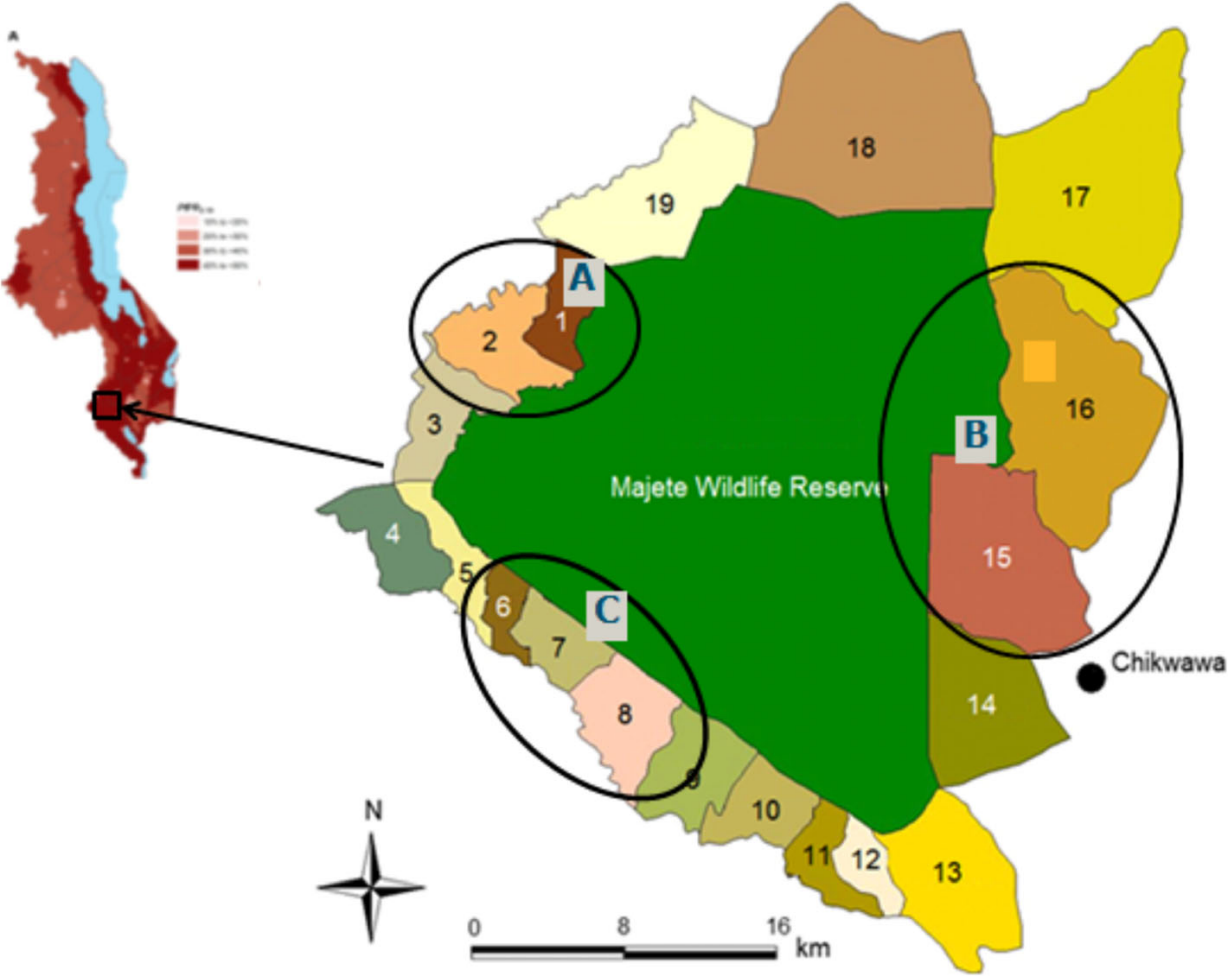
Map of MMP focal areas. The green area represents the Majete Wildlife Reserve which is surrounded by community bases organizations (CBO’s). Focal area A consists of CBO 1 and 2, focal area B consists of CBO 15 and 16 and focal area C consists of CBO 6, 7 and 8. Source map: Majete Malaria Project. (Map source from ‘Adaptive geostatistical sampling enables efficient identification of malaria hotspots in repeatedcross-sectional surveys in rural Malawi’ [14])

This was a cross-sectional health facility survey using quantitative methods and was conducted in Chikhwawa district in Southern Malawi. Transmission of *Plasmodium falciparum* in Chikhwawa occurs throughout the year but peaks during the rainy season - December to April [13]. This survey was done in June and July in 2017, under Majete Malaria Project (MMP) in three defined “focal areas” A, B and C (Fig. 1). Details on the funding of MMP can be found in the declarations section. These three focal areas were selected based on the presence of an existing or developing community-based livelihood centre. MMP encourages community engagement and participation in disease control by using trained community volunteers who conduct community workshops on malaria in their villages. Workshops aim to increase malaria awareness and promote positive health behaviour [15].

Health centres within MMP focal areas were included in the study. Health centres provide primary health care within a catchment population. Health centres are usually staffed by at least one clinician (clinical officer or medical assistant) and two nurse-midwife technicians. A clinical officer undergoes 3 years of clinical training while a medical assistant undergoes 2 years. A nurse-midwife technician does 3 years of training. The primary healthcare services provided in health centres include management of outpatient conditions, minor surgical procedures, antenatal clinics, maternity and postnatal care and childhood vaccinations. Surgical patients requiring major procedures and patients needing admission are referred to a district hospital where secondary healthcare is provided.

### Study population

The study population includes children aged 6 months to 10 years old presenting with a history of fever at the health centre during the data collection period. Children with features of severe illness were excluded. For the health facilities, facility officers in charge were interviewed on availability of medical supplies, equipment and staff using a structured questionnaire.

### Operational definitions

Adherence to malaria case management guideline in primary healthcare settings was defined as: performing an mRDT test for children with reported fever; prescribing first-line antimalarial drugs to all children with a fever and positive malaria test; not prescribing antimalarial drugs to children with a negative mRDT result; and prescribing drugs based on the weight of the child. These definitions are consistent with the current *Malawi Standard Treatment Guidelines* (MSTG) and *WHO malaria guidelines* [2, 8]. Recommended first-line ACT in Malawi at the time of the survey was artemether- lumefantrine (AL). Prompt health care seeking behaviour was defined as presenting at the health centre within 24 h of fever onset as per WHO recommendation [2].

### Data collection

The research team was present for in total 4 working days at each health facility. We assessed the following health centres: Kaphichira, Kakoma, Chapananga, Misomali, Chithumba and Majete. Questionnaires used for the exit interviews were used from a previous study by the MMP [14].

#### Caregiver interview

Exit interviews were conducted with guardians of eligible children. The guardians were interviewed using a structured questionnaire on an electronic tablet. During the exit interviews, sociodemographic details were documented. Additionally, we collected data on attendance of community workshops, time taken travelling to the health facility and duration of symptoms before presenting at the facility. An estimation of travel time was based on the village the child and the guardian travelled from. Guardians were asked for approximated travel time from their home to the facility. This was crosschecked with the health workers at the facility if the duration of travel time based on the mode of transport used was deemed reasonable. Google maps was used when the investigator or a staff member from the clinic could not make an estimation on travel time. The child’s health passport, which includes the patients’ medical records, was reviewed to collect documented symptoms, axillary temperature, weight, conjunctival pallor, mRDT result and prescribed treatment.

#### Patient clinical assessment

A clinical assessment of the patient was repeated by LK and AEC who are both clinicians. The assessment included weighing the children using a calibrated digital scale and measuring an axillary temperature using a digital electronic thermometer. The investigators clinically re-evaluated presence of anaemia by examining the conjunctiva and palms for pallor. Malaria test result was obtained from the health passport and not performed by the researchers.

#### Health workers’ interview

Availability of staff, medical equipment, essential drugs and facility supervision were evaluated through an interview with the officer in-charge of the facility. Stock outs were evaluated by assessing the specific mRDT, AL and paracetamol stock cards in prior three-month records. Availability and functionality of the following essential equipment was recorded: weighing scales, thermometers, hemocues and microscope. We evaluated completeness of outpatient (OPD) consultations, mRDT and AL registers containing records of the previous 3 months prior to the visit.

### Data processing and analysis

To protect patient safety all personal data were removed during data transcription from the source document to tablet and into the database. Data in the tablet were checked for completeness and then sent to a remote server via a wireless internet connection. Data were then downloaded and analysed using STATA version 14.0 and SPSS version 20.0. The main outcomes were adherence to malaria case management guidelines and promptness to seek care. We used univariate analysis to report frequencies and proportions for adherence to malaria case management guidelines. Promptness to seek care was categorised into: within 24 h of onset of symptoms and after 24 h. For promptness to seek care within 24 h, chi square tests were used in the bivariate analysis to check differences in proportions for categorical variables and promptness to seek care. Covariates were included in the logistic regression analysis when *p*-value was < 0.05. For promptness to seek care, the following variables were determined a priori based on previous studies and expected associations: distance to health facility, source of income, highest level of education and health animator workshop attendance. Logistic regression assesses potential predicting factors in promptness to seek for care.

## Results

### Health facilities

One health centre was a private facility, one was a Christian health association of Malawi (CHAM), and the other four were government facilities (Table 1). Each health centre had at least two health service providers available and several health surveillance assistants. Kapichira had a clinical officer as the officer-in-charge. All other facilities had a medical assistant as the officer-in-charge. Chapananga had the highest number of children sampled (*N* = 95) while Kapichira had the lowest (*N* = 20) see Table 1.

**Table 1:** Health facility characteristics

Health center	Focal area	Type of health center	Number of children sampled	In-charge	NMT	HSA
Kapichira	B	Private	20	Clinical officer	1	6
Kakoma	C	Government	63	Medical assistant	1	13
Chapananga	C	Government	86	Medical assistant	1	7
Misomali	C	CHAM	26	Medical assistant	1	4
Chithumba	A	Government	37	Medical assistant	1	2
Majete	A	Government	24	Medical assistant	1	2

*CHAM* Christian Health Association of Malawi, *HSA* health surveillance assistant, *NMT* nurse midwife technician

### Summary characteristics and study participants

Out of 265 children screened, nine were excluded due to presence of danger signs. The danger sign reported was convulsion. Of the 256 children enrolled the median age was 3.2 years (interquartile range 1.5–5.7 years) and half of them were female (Table 2). Most patients (82.8%) came to the facility with their mother as their guardian. In total, 46.1% of guardians had attended a health animator workshop at least once. The majority of guardians (77%) had attended some school, with a few (8.8%) attending secondary school. Subsistence farming was the main source of income for most households (44.1%), although some households relied on commercial farming (33.2%). In total 143 children (55.9%) had a temperature above 37.5 degrees Celsius when re-measured by the research team. All children reported other symptoms as well: 34 reported diarrhoea (13.3%), 89 vomiting (34.8%), 112 headache (43.8%), 142 cough (55.5%), 40 dyspnoea (15.6%), 27 abdominal pain (10.5%), 13 rash (5%) and 10 ear pain (3.8%). In total 4 (1.6% (95% CI 0.4–3.1)) guardians of children reported their child to have had malaria in the past 2 weeks prior to the consultation. In this group mRDT had been performed at the previous consultation and AL treatment prescribed and completed. For 30 children (11.7 95% CI [8.6–16.0]), the clinician had enquired about a malaria history within the previous 2 weeks. The mean waiting time before consultation after reaching the health facility was 68 min (SD = 55) according to guardian’s estimation. After consultation the mean time before receiving drugs at the dispensary was 28 min (SD = 89).

**Table 2:** Socio demographic characteristics of children and their guardians

Characteristic	Frequencies
Male	128 (50%)
6 months to 5 years	75 (29.3%)
5 years to 10 years	181 (70.7%)
Guardian	
Mother	212 (82.8%)
Father	21 (8.2%)
Other	23 (8.9%)
Guardian level of education	
None	57 (22.3%)
Some primary	149 (58.2%)
Completed primary or more	49 (19.1%)
Average hours taken traveling time to facility (SD)	1.2 (1.15 SDS)
Travel mode	
Walking	206 (80.5%)
Bicycle	47 (18.0%)
Vehicle	4 (1.6%)
Household source of income	
Subsistence Farming	113 (44.1%)
Commercial farming	85 (33.2%)
No employment	2 (0.8%)
Other employment^a^	56 (21.9%)

*SD* standard deviation

^a^Shop owner, temporary employment, NGO employment, teacher

Pallor was assessed in 168 (65.7%) patients. In all 169 (66.0%) cases of confirmed malaria, haemoglobin level was not measured.

### Access and prompt health care seeking behaviour

Overall, 146 (57%) guardians of children reported to have presented within 24 h of onset of symptoms; 31 guardians (12%) presented after 48 h. Most guardians *(*80.5%) walked to the facilities. The mean time for all modes of transport, to travel to the health centre was 1.2 h (SD = 1.1). Mean time for guardians who walked, cycled, travelled by vehicle or bicycle taxi was respectively 1.3 h (SD = 0.1), 1 h (SD = 0.7), 0.7 h (SD = 0.2), 1.2 h (SD = 0.6). No statistically significant associations were found between the a priori selected covariates and the main outcome in bivariate analysis. Therefore, these results are not reported here. Level of education (*p* = 0.230), health animator workshop attendance (*p* = 0.237), source of income of household *p* = 0.589) and distance in hours to the health facility (*p* = 0.983) were not significantly associated with prompt care-seeking (within 24 h) in the logistic regression analysis. Only age was significantly associated with seeking care within 24 h of symptom onset in the logistic regression analysis: children aged 5–10 years were less likely to present promptly within 24 h of fever onset than children aged below 5 years of age with (*p*-value < 0.001; OR = 0.397, (95% CI [0.227–0.695]), see Table 3.

**Table 3:** Promptness to seek care among guardians of children with fever and associated factors

	Odds ratio	95% confidence interval	*P*-value
Health animator workshop attendance			
Yes	[reference]		
No	1.375	0.811–2.330	0.237
Level of education			
Some primary	[reference]		
None	0.682	0.357–1.302	0.246
Primary or more	1.354	0.670–2.737	0.398
Source of income			
Other employment	[reference]		
Farming	1.178	0.630–2.203	0.608
Distance to health facility in hours	1.001	0.779–1.253	0.995
Age			
Under 5 years	[reference]		
5–10 years	0.397	0.227–0.695	0.001

### Health facility factors: availability of medical equipment and drugs

Five out of the six health facilities had analogue weighing scales for both adults and children. Functional thermometers were available in all health facilities. Only one health facility had hemocue and cuvettes. None of the facilities provided microscopy for malaria diagnosis. Only one facility had a functional ambulance for referral to a secondary healthcare facility. None of the facilities had in-patient admission capacity.

There were stock outs of paracetamol in two facilities recorded in stock cards within the 3 months prior to the survey. AL stock outs of one or more AL blister packs were reported in five of the six facilities in the 3 months prior to the survey. Stock outs of AL 1 × 6, 2 × 6, 3 × 6 and 4 × 6 on average occurred for 11, 20, 15 and 13 days, respectively. AL blister packs were never completely out of stock concurrently. In situations where one blister pack type was out of stock, the clinician used other available blister packs. mRDT kits were recorded out of stock for 16 days at the time of the survey in one health centre. Artesunate injectable was available in all health facilities and not reported to have been out of stock in the previous 3 months. Oral quinine was not available at any of the 6 health centres. Five out of six (83%) facilities provided bed nets.

### Health workers practices

**Fig. 2:**
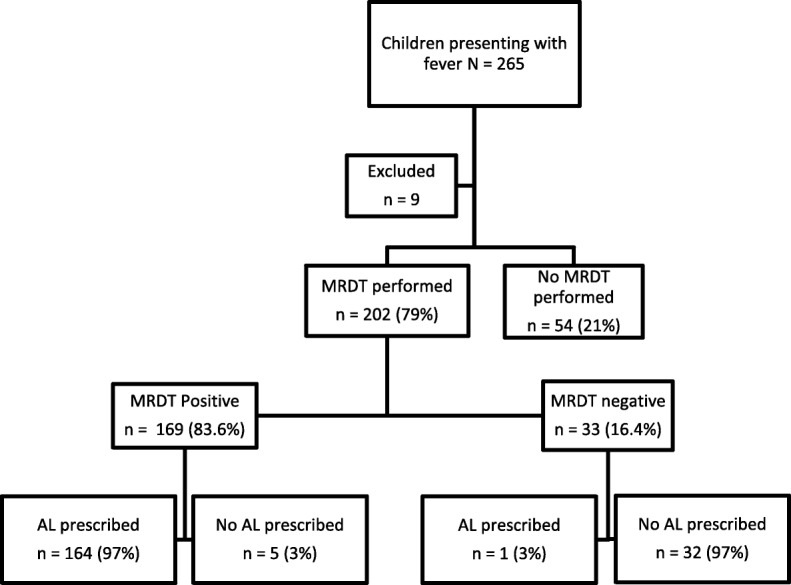
MRDT testing and AL prescription practices

All facilities had a copy of the *Malawi Standard Treatment Guidelines.* Weight and temperature were measured in 64.5 and 24.2% of the children, respectively. In total 79% (95% CI [73.7–84.4]) of children who presented at the health centres with fever received an mRDT test (Fig. 2). For the 54 children not tested, 56% were due to an mRDT stock out. No clear reasons were provided in the other children not tested. Adherence to positive mRDT was 99.4% (95% CI [98.1–100]).

Adherence to negative mRDT results was 97% (95% CI [88.9–100]). Sixty-six percent (95% CI [60.5–71.7]) of all children who received an mRDT had a positive mRDT. Weight-based dosing was correct in 94.3% (95% CI [89.4–98.1]) in weight category 5–15 kg, in 88.5% (95% CI [86.3–96.1] in 15–25 kg) and in 100% in 25–35 kg. In total 6 (3.6%) children were under-dosed and 7 (4.2%) were overdosed. In one health centre, complete stock out of mRDT occurred during the visit of the research team. All patients presenting at this specific facility with fever were prescribed co-trimoxazole then referred for malaria testing at the district hospital.

### Health information system

All patients presenting at a health facility and seen by a clinician are recorded in the outpatient register. All mRDTs performed and results are documented in the mRDT register. Monthly summaries of total number of OPD consultations, mRDTs performed, AL prescribed (and which type of AL) are sent to the District Health Office (DHO). In three health centres, registers were incomplete and with no monthly summaries. In the other three facilities the registers daily reports on OPD attendance, mRDT testing and AL prescription were complete. Overall, including the incomplete registers, in five health centres there was a mismatch in the number of MRDTs performed and the expected quantity of AL dispensed (Table 4).

**Table 4:** Summaries of mRDT-, AL-, and OPD registers in the previous 3 months prior to the health facility assessment

	Total under 5 OPD treated for malaria *(OPD and AL register)*	Total under 5 OPD treated for confirmed malaria *(OPD, mRDT, AL register)*
Kapichira	425	108
Kakoma	3837	904
Chapananga	2203	2203
Misomali	612	809
Chithumba	193	899
Majete	148	141

## Discussion

This study highlights the challenges affecting access to prompt and effective malaria case management in health facilities within a rural community. Challenges were prompt care seeking, stock outs of mRDTs and AL and health worker practices in case of stock outs.

Some guardians delayed seeking care for their children within 24 h after onset of fever. This finding is similar to the 40.9% that did not present within 24 h in the 2015 study in Chikhwawa [14]. Ewing et al. reported that within Chikhwawa most households had a ‘wait and see’ policy when the child had fever [5]. Only when fever persisted and serious illness was perceived, guardians would seek hospital care. Studies in Malawi and Tanzania, found a lack of guardians’ knowledge on malaria and fever to contribute to delayed care seeking behaviour [14, 16]. Our study did not find a statistically significant association between prompt care-seeking and the level of education, distance to the health facility, health animator workshops attendance or source of income of the guardian. The lack of association between level of education, socio-economic status and care-seeking was contrary to other findings in literature which have shown an improvement in health promotion when increasing malaria awareness [17–19]. Also education and distance to a facility were found to be of influence in health care seeking in other studies [5, 20–22]. Further qualitative analysis is needed to identify other factors that influence delayed care seeking behaviour. Prompt care-seeking by guardians for children aged 5–10 years was lower than in the category under 5 years. A qualitative study in Nigeria showed mother’s beliefs towards physician’s approach to illness also played a role in seeking care for their under-5 year old child [23]. Guardians may have easily noticed fever in their under-fives since young children are mostly at home and close to their guardians. Improving prompt care seeking would contribute to better access and efficacy of malaria treatment.

Stock outs of malaria diagnostics and treatment continue to be a challenge to accurate malaria case management in Chikhwawa district. Lack of testing was largely due to mRDT stock outs. Incomplete mRDT and AL registers can influence drug and diagnostics supply chain negatively. AL and mRDT supplies are calculated based on the registered numbers. Stock outs can influence health workers to prescribe unrecommended antimalarial drugs or antibiotics [24, 25]. One health centre had complete stock out of mRDT and AL. Due to strict adherence to the malaria guidelines when mRDTs were out of stock, none of the children presenting with fever were presumptively treated with ACT. This inadequate malaria treatment causes a risk of severe disease and increases the risk of transmission. A Cochrane review assessed the beneficial effect of mRDT testing versus clinical diagnosis and showed that mRDT testing is beneficial for the mRDT negative cases who might have otherwise received an ACT [26].

The adherence to positive and negative mRDT results was high. These results are similar to the health facility survey in Malawi performed by Namuyinga et al. in 2015 [12]. Similar numbers were found in a health facility survey in Uganda in 2016 assessing adherence to negative mRDT results [27]. The percentage of inappropriately prescribed AL is much lower than the reported 31% in the health facility survey performed in Malawi in 2011 by Steinhardt et al. [10]. A similar cross sectional health facility survey performed in Chikhwawa in 2015 showed inappropriate dosing of AL in 24% of cases [14]. The low number of health workers per health facility creates a high workload and influences health workers’ practices [28].

There are several limitations to this study. The study was performed in a small number of health centres in a small scale-sized geographical area. However results of the health facility assessment might be applicable on a larger scale to other parts of rural sub-Saharan Africa [10]. The data for the exit interviews were obtained from guardians posing a possibility of social desirability bias. Presence of the research team at the health facilities might have influenced health worker practices and might have enhanced adherence to mRDT result and prescription practices. However, we tried to minimize this Hawthorne effect through partial disclosure of the main purpose of the study to the officers- in-charge. Moreover, retrospective evaluation of the AL, mRDT and OPD registers indicated appropriate malaria case management practices.

## Conclusion

This study showed that adherence to malaria case management guidelines was high in primary health facilities in Chikhwawa district. However, prompt care seeking and stock outs remain challenges to prompt and effective malaria case management. Continuation of increasing malaria knowledge and awareness is needed to assure early care seeking. Further qualitative work is required to determine and address factors which reduce prompt health care seeking behaviour and health worker practices to further improve access to prompt and effective malaria case management.

## Data Availability

Data will be made available on reasonable request to the first author: L. Klootwijk.

## Abbreviations

ACT: Artemisinin-based combination therapy
AL: Artemether-lumefantrine
CHAM: Christian Health Association Malawi
COMREC: University of Malawi College of Medicine Research and Ethics
DHO: District health officer
MMP: Majete Malaria Project
mRDT: Malaria rapid diagnostic test
MSTG: Malawian Standard Treatment Guidelines
OPD: Outpatient department
WHO: World Health Organisation
